# Supplementary material for: Systemic hypoxia inhibits T cell response by limiting mitobiogenesis via matrix substrate-level phosphorylation arrest
Source: eLife. 2020 Nov 23;9:e56612. doi: 10.7554/eLife.56612 (PMC7728436; doi:10.7554/eLife.56612)
Supplement: Supplementary file 1. [file elife-56612-supp1.docx]

| Category | ATP Domain | GTP Domain |
| --- | --- | --- |
| Purines, Pyrimidines Synthesis | Paics, Mthfd1l, Fpgs, | - |
| Protein Import/maturation/assembly | Hspd1, Hspa9, Afg3l2, Bcs1l, Trap1, Timm44, | - |
| mTranslation | Aars2, Rars2, Dars2, Cars2, Qrsl1, Ears2, Gatb, Gatc, Hars2, Iars2, Lars2, Mars2, Fars2, Pars2, Sars2, Trmu, Trit1, Tars2, Wars2, Yars2, Vars2 | Noa1, Mtg1, Mtif2, Tufm, Guf1, Gtpbp3, Gtpbp6, Gtpbp10, Gfm1, Gfm2, Eral1 |
| mRNA Transcription/ Processing | Ddx28, Supv3l1, Mtpap, | - |
| mDNA Replication | Nme4, Nme6, Peo1, Dguok, | - |
| Iron Sulfur Cluster | - | Mocs1 |
| Protein Degradation | Lonp1, Lace1, Ide, Clpx, | - |
| Metabolism | Acly, Coasy, Nadk2, Acsf1, Acsf2, Acsf3, Mmab, Glud1, Flad1, Coq8a, Coq8b, Bckdk | - |
| Transport | Abcb10, Abcb7 | - |
| Mitochondrial morphology | Atad3a | - |
| Urea Cycle | Cps1 | - |

| **Supplementary file 1: Matrix protein annotated by GO with either ATP or GTP domains** |
| --- |
