# Supplementary material for: Systemic hypoxia inhibits T cell response by limiting mitobiogenesis via matrix substrate-level phosphorylation arrest
Source: eLife. 2020 Nov 23;9:e56612. doi: 10.7554/eLife.56612 (PMC7728436; doi:10.7554/eLife.56612)
Supplement: Transparent reporting form [file elife-56612-transrepform.docx]

***eLife’s* transparent reporting form**

We encourage authors to provide detailed information *within their submission* to facilitate the interpretation and replication of experiments. Authors can upload supporting documentation to indicate the use of appropriate reporting guidelines for health-related research (see [EQUATOR Network](http://www.equator-network.org/%20)), life science research (see the [BioSharing Information Resource](https://biosharing.org/)), or the [ARRIVE guidelines](http://www.plosbiology.org/article/info:doi/10.1371/journal.pbio.1000412) for reporting work involving animal research. Where applicable, authors should refer to any relevant reporting standards documents in this form.

If you have any questions, please consult our Journal Policies and/or contact us: [editorial@elifesciences.org](mailto:editorial@elifesciences.org).

**Sample-size estimation**

- You should state whether an appropriate sample size was computed when the study was being designed
- You should state the statistical method of sample size computation and any required assumptions
- If no explicit power analysis was used, you should describe how you decided what sample (replicate) size (number) to use

Please outline where this information can be found within the submission (e.g., sections or figure legends), or explain why this information doesn’t apply to your submission:

In vivo studies- We conducted the experiment under two main assumptions: a. we expect to observe a very large effect i.e. effect of 50% in respect to control. b. effect is expected to be observed only in one direction (increase or decrease in T cell response). Given these assumptions and the small number of groups in the experiment we designed the experiments to have 5-6 mice per group.

In vitro studies – Cell trace and activation experiments were design to observe major inhibitory effect i.e. more than 50% we therefore used between 5-6 replicates in each well sufficient to reach significance in a standard T test

rtPCR- 10 replicates were used to allow for technical failure and to observe differences over 30%

Big-Data analysis – a. proteomics - 3 replicates were used to capture only major change in mitochondrial proteins ubiquitination levels b. metabolomics 5 replicates were used in each group to capture major shift in metabolites

**Replicates**

- You should report how often each experiment was performed
- You should include a definition of biological versus technical replication
- The data obtained should be provided and sufficient information should be provided to indicate the number of independent biological and/or technical replicates
- If you encountered any outliers, you should describe how these were handled
- Criteria for exclusion/inclusion of data should be clearly stated
- High-throughput sequence data should be uploaded before submission, with a private link for reviewers provided (these are available from both GEO and ArrayExpress)

Please outline where this information can be found within the submission (e.g., sections or figure legends), or explain why this information doesn’t apply to your submission:

All experiments were conducted 3 times. Data regarding the design of the experiment and the number and type of replicates could be found in the figure legend and the methods section. Outliers were removed only when technical issue was observed e.g. bubbles in a sea horse well

**Statistical reporting**

- Statistical analysis methods should be described and justified
- Raw data should be presented in figures whenever informative to do so (typically when N per group is less than 10)
- For each experiment, you should identify the statistical tests used, exact values of N, definitions of center, methods of multiple test correction, and dispersion and precision measures (e.g., mean, median, SD, SEM, confidence intervals; and, for the major substantive results, a measure of effect size (e.g., Pearson's r, Cohen's d)
- Report exact p-values wherever possible alongside the summary statistics and 95% confidence intervals. These should be reported for all key questions and not only when the p-value is less than 0.05.

Please outline where this information can be found within the submission (e.g., sections or figure legends), or explain why this information doesn’t apply to your submission:

We used non-parametric T test to identify significance as most of the experiments were designed to test for very large differences between two to three groups (Typically the direction of the effect was already anticipated). In Big Data analysis we used FDR 0.05 to avoid false positives.

P- values error and replicates are reported within the legends

(For large datasets, or papers with a very large number of statistical tests, you may upload a single table file with tests, Ns, etc., with reference to sections in the manuscript.)

**Group allocation**

- Indicate how samples were allocated into experimental groups (in the case of clinical studies, please specify allocation to treatment method); if randomization was used, please also state if restricted randomization was applied
- Indicate if masking was used during group allocation, data collection and/or data analysis

Please outline where this information can be found within the submission (e.g., sections or figure legends), or explain why this information doesn’t apply to your submission:

Mice were allocated randomly from same colony and when possible cage.

Cells were harvested from the same source and when required pooled from a number of mice.

**Additional data files (“source data”)**

- We encourage you to upload relevant additional data files, such as numerical data that are represented as a graph in a figure, or as a summary table
- Where provided, these should be in the most useful format, and they can be uploaded as “Source data” files linked to a main figure or table
- Include model definition files including the full list of parameters used
- Include code used for data analysis (e.g., R, MatLab)
- Avoid stating that data files are “available upon request”

Please indicate the figures or tables for which source data files have been provided:

Big-Data sets were provided as links to third party servers as part of the submission
